# A Resume of the Study of Saliva1Read before the Garretson Dental Society, Philadelphia November 21, 1904.

**Published:** 1905-12

**Authors:** Samuel Doskow


### A RESUME OF THE STUDY OF SALIVA.[1]

BY SAMUEL DOSKOW, D.D.S.

Mr. President and Gentlemen: In the human economy there are, in connection with the general apparatus for the carrying on of the metabolic process, two systems that play a great part in the proper performance of that process. One, occupied in the formation of substances that do not preexist in the blood, and are employed for the purpose of serving some ulterior purpose in the economy, and which bears the name of *secretion.* To this class belong such substances as saliva, bile, pancreatic juice, etc., etc. The other tends to rid the economy of waste and injurious materials that are called excretions. The constituents of this class are urine, feces, sweat, etc.

Under normal circumstances, *i. e.,* when the food ingested is the proper amount, proper kind, and properly assimilated, both the secretions and excretions will be in proportion as regards both the quantity and composition. But when the normal is not realized, either through some fault on the part of the food or through some impairment of the digestive apparatus, the functions of both the secretory and excretory processes will be interfered with.

---

[1] Read before the Garretson Dental Society, Philadelphia November 21, 1904.

Hence, if on examinations of the secretions or excretions, they are found to deviate from the normal, the conclusion of the existence of a defect in the metabolism of the economy is a natural corollary.

In a paper read before the Odontographic Society of Chicago, February 16th, 1903, Prof. Edward C. Kirk likens the human economy to that of a furnace; that when there is a lack of proportion between draught and fuel, the energy produced by it will not be the kind nor amount desired for the accomplishment of the work intended by it, also that the products and waste of the combustion from that furnace are good indicators in the hands of the chemist for the determination of its condition.

In the body, the alimentary tract plays the part of the furnace; the fuel is represented in the food ingested ; the draught is furnished by the secretions ; the parts absorbed by the blood represent the energy created; and the waste is found in the excretions.

This analogy falls short in this one respect, that while in the mechanical furnace the furnace itself has no influence over the place of supply of the draught, in the human economy this influence is brought about in an indirect way. For instance, should the food ingested be in some way inimical to the lining of the stomach, the effect will be that the secretions of the pyloric and cardiac glands of that organ will be greatly modified. A like effect may in turn be brought to bear on some of the larger glands. The effect then becomes double; the glands affected will produce a secretion that is not fully capable of performing the function intended, and it in turn further disables the activity of the digestive tract.

The above illustrations give proof to the statement made in the earlier part of this paper that the compositions of the secretions and excretions of the body are determining factors of the metabolic activity of the economy whether it be normal or not.

Having established the proof that the secretions and excretions are reliable means for the determination of the normal or abnormal states of the economy, I shall now devote the rest of my paper to one secretion of the body, although it has not been regarded heretofore of diagnostic value; still from the part it plays, its relation to the general metabolic activity, and especially in view of some researches that have been recently made, I think it is worthy of consideration. This secretion is saliva.

In looking over the history of the subject, we find that the study of it dates as far back as 1838, when Dr. A. Donné published a treatise considering saliva as a means for diagnosing certain diseases of the stomach. He was followed by others, but it was not until 1884 that the first treatise of importance came to light. I refer to the work of Dr. Binet, entitled "Etude sur la Sueur et la Salive." He wanted to know what were the substances contained in sweat and saliva under normal conditions; also the relations existing between them. In 1900, Dr. Joseph P. Michaels, of Paris, presented before the Third International Dental Congress a large thesis—Sialo Semeiology—of careful systematic observations made during a period of six years. He introduced the use of the micro-polariscope for its examination. The advantage gained by it is that the light is changed from a ray radiating in all planes to one that radiates only in one plane as if passing through a grating. By that means he was able to detect crystalline substances that are not detected by ordinary light. About this time Prof. Edward C. Kirk, of this city, took up the study of this subject. He was at once confronted with the difficulty that the salts were very minute owing to the colloid substances in the saliva, and that although they presented a beautiful view for observation from the point of view of the antiquarian they did not present sufficient characteristics for the determination of their exact composition. To overcome this difficulty he determined to remove the colloid substances from the saliva so that they should not interfere with the proper crystallization of the salts. This was accomplished by means of a dialyzer especially constructed for this purpose. As a result of this, a definite work was done on the composition of saliva in relation to erosion: "The Clinical and Chemical Study of a Case of Erosion, March, 1902;" its diagnostic value: "The Saliva as an Index of Faulty Metabolism, February, 1903;" its relation to caries: "The Predisposing Factor in Dental Caries, March, 1903."

Saliva is a mixed fluid that is secreted into the mouth from various glands situated in the proximity of the oral cavity through their respective ducts. It assists in articulation and deglutition, and plays an important rôle in the first stage of digestion. It converts the starches into sugars; first into dextrin and maltose, later converting the dextrin into maltose. According to Michaels, normal saliva should be transparent, clear, bluish in color, possessing neither taste nor odor, neutral in reaction, diastasic, containing sodium chlorides, but no polarizable salts.

Regarding the above as a standard guide we determine that any deviation from it means abnormal saliva. From observations made by Michaels it was found that certain characteristics of the saliva exist under certain metabolic conditions. It was also found that those characteristics are constant under similar diathetic conditions in different individuals. It was further observed by Prof. Kirk that the development of a certain abnormal metabolic condition is in a graded scale beginning with the element most abundant and easier dispensed with, and further, that in the treatment of that condition for the restoration of the economy to the normal the replacement of the elements lost is in the reverse order of the graded scale. This establishes beyond doubt the reliability of the saliva as a means for the determination of the different oscillations in the economy when external relations are not in accord with internal relations in whatever phase it may be manifested.

The disposition of the economy towards the gradual development of abnormal metabolic conditions, thereby lessening its vital resistance to the attack of specific disease, known as diathesis, is subdivided into two distinct heads: That condition in which the oxidation process is over-active, resulting in a decrease of organic acidity with a corresponding increase in saline chlorides excreted by the economy, is known as hypoacidity. This condition renders the body favorable for the development of various contagious diseases, such as scrofula, tuberculosis and syphilis. Fortunately this condition is not prevailing, or else the fate of the civilized races would be in as sorrowful a state as some of the Indian tribes on our borders, who resort to the adoption or acquisition of white children, so that the extinction of their tribes may be thereby prevented. The other diathetic condition is the reverse of the former. It is characterized by an under or suboxidation in the metabolic process; a general slowness in the biochemical changes ; the gradual deposition of acid salts in all the articular tissues, and especially in those most active ; the increase of organic acidity as a consequence. It leads to the development of gout, rheumatism, sclerosis, etc. According to Gautrelet and others, if the amount of waste products distributed through the blood-plasma and its constant increase be taken into account, the blood in this condition should be regarded as decidedly acid in reaction. It approaches senility. Many individuals are found to-day that although young in age are really old as far as their metabolic activity is concerned. This diathesis is termed hyperacidity.

The composition of the saliva under the different diathetic conditions varies with the degree or period of the diathesis. In the hypoacid diathesis, the saliva, according to Michaels, is usually abundant in quantity, opalescent, of fluid consistence, white, insipid, of nauseating odor, actively fermentative, alkaline in reaction and contains crystalline ammoniacal salts. Caries of the teeth is most abundant in this diathesis. Our experience with this kind of saliva is very limited, and I shall therefore leave it and consider more fully the saliva of the hyperacid diathesis.

As stated above, hyperacidity is characteristic by a slowness in the biochemical changes of metabolic activity, resulting in the deposition of the various crystalline salts throughout the course of the blood plasma. The casting off of the various elements from the economy is in a slow, gradual way, beginning with the element most abundant and easier dispensed with. The elements affected, and which are at the basis of the chemical construction of the body, are sodium, calcium, and magnesium. The acids connected with the precipitation of these elements that afterwards are dialyzed through the gladular structures and appear in the saliva are: phosphoric, lactic and oxalic acids. The first installment of salts that gives the indication of the existence of the diathesis in question is the phosphates of sodium, first appearing as neutral phosphates and later as the acid-sodium-phosphate. The appearance of this salt in saliva is noteworthy, inasmuch as we know that this salt in small quantity is a normal constituent of the urine. The presence of a salt that is a normal constituent of the urine has no place in normal saliva. The action of the renal epithelium upon the blood when it reaches the kidneys produces the reaction forming this salt, thereby giving the urine its acid reaction, while the blood remains alkaline in reaction.

$$Na_2HPO_4+H_2CO_3=NaH_2PO_4+NaHCO_3.$$

With the advent of the diathesis, when the supply of sodium has been largely depleted the calcium is affected, and we will first get the combination of sodium and calcium and later pure crystals of the calcium acid phosphate. The condition of the teeth at this stage depends upon whether they were affected by caries prior to the development of this stage. Usually there is an absence of caries, for when the saliva reaches an acidity of this strength the development of bacteria is almost impossible. But should the teeth have been affected by caries prior to the development of this diathesis they will present certain characteristics that in themselves are determining factors. The carious matter assumes a dark brown color, of a semi-hard texture, and when the bur is applied it crumbles away like dried rotten wood. Unlike teeth that are in an acid medium, they are almost devoid of sensation. The destruction of the teeth does not seem to cease with the cessation of the development of caries; on the contrary, destruction proceeds, but from a totally different cause. They are, so to say, dissolved out.

A striking illustration of this kind presented itself at the clinic of the University of Pennsylvania, and upon careful study much valuable information was derived from it. The patient was a young man of about twenty-five years of age. Almost every tooth in his mouth was decayed. The condition of the decay was like that described. On examination of his saliva it was found to contain very large crystals and a large quantity of acid-sodium-phosphate. He showed every condition of approaching old age, and judging from the amount of phosphates in his saliva it was concluded that he was a phosphorous diabetic. He was instructed as regards habit and diet and phosphorus was administered to him in various ways and in varying doses. In a comparatively short time he showed marked signs of improvement. Unfortunately we were not able to follow this case to completion, but we learned from the student who had him in charge for the treatment of the disorders about the teeth, that he was following the instructions as outlined to him in the laboratory and is faring well.

Following up this stage in regular order come the lactates. We first have the lactates of sodium, then of calcium and magnesium. Later come the double lactates of sodium and calcium. Then we have the combination of lactates and phosphates, such as the lacto-phosphates of calcium and lacto-phosphates of magnesium. These crystals are very rich in color when viewed by porlarized light, and possess definite outlines distinct from any family of salts. The action upon the teeth is very marked. It is here that we have that class of erosion that was named by Prof. Kirk as the general erosion in contradistinction to the localized erosion that is caused by the sodium-acid-phosphate from some of the buccal glands. A systematic study of this subject had been presented by Prof. Kirk before the Second District Dental Society of the State of New York in the paper referred to above.

The third stage in the development of this diathesis is the appearance of the oxalates. So far we have only met with the oxalate of sodium. The appearance of this salt is usually a signal of nervous debility, and in many cases complete derangement of the nervous system. There are many cases to which I can refer that were analyzed at the laboratory, diagnosis determined, and for whom valuable aid was rendered towards restoring them to the normal. I shall, however, quote only one case, that I may say was nipped in the bud and which is under my observation almost every day. The case is that of a young man of about twenty-two, who complained that his appetite was not as he thought it should be and that he feels as if something is lacking. Examination was made of his saliva and urine, as is done in every case. It was found that in addition to the few diacid-phosphate crystals he had a large number of the sodium oxalates—thus proving that he was drained of his phosphates to a large extent and that his system was manufacturing oxalic acid and assisting the phosphoric acid in its destructive activity as well as acting as a toxic irritant. A diet was prescribed to him, barring all carbohydrates and such vegetables as help in the formation of oxalic acid. In addition to this he was ordered to drink hot water as many times during the day as he was able to manage and a tonic of phosphorus and arsenic in small doses, in the form of a pill, taken three times a day. He continued taking the tonic for about three months but he is still adhering to his diet. He has since gained in health, his weight has increased and he assured me many times upon questioning him in regard to it that he could not expect to feel better.

The oxalates appear sometimes as double oxalates of sodium and calcium, while the oxalate of magnesia is often found in urine. When this occurs the indications are that the individual is sliding down a steep slope, and unless efforts are made to prevent him from landing in the pit, serious complications will follow.

I acknowledge my indebtedness to Prof. Edward C. Kirk, who first advised me to follow this line of study. His personal instruction and direction in the laboratory helped to inculcate into my mind the comprehension of the vast scope of this work and to what extent mankind in general could be benefited by it; also, that there is a domain in dentistry in addition to that of filling teeth and the making of artificial appliances for the restoration of the lost organs, important as that may be. The study of prophylaxis could not in any way be undertaken or understood, unless the condition of the system is studied in a systematic and scientific manner.

